# TruVox Web-Based Software for Vocal Pitch Training in Transgender Women: Development and Single-Session Evaluations

**DOI:** 10.2196/73841

**Published:** 2025-09-15

**Authors:** Sam R Weese, Mary E Wilkens, Om Jadhav, Xiangyi Wang, Ansh Bhanushali, Tyler DiLoreto, Reyna Kozel, Renee L Gustin, Tara McAllister, Victoria Sue McKenna, Vesna Dominika Novak

**Affiliations:** 1 University of Cincinnati Cincinnati, OH United States; 2 Department of Communicative Sciences and Disorders New York University New York, NY United States; 3 School of Communication Sciences and Disorders University of Central Florida Orlando United States

**Keywords:** transgender health, exercise technologies, serious games, voice training, usability, user experience, mobile phone

## Abstract

**Background:**

Transgender people often experience distress due to a mismatch between their gender and the way their voice is perceived (eg, transgender women with low pitch), which significantly reduces their mental health and quality of life. This is especially a problem for transfeminine people and can be reduced with gender-affirming voice training (GAVT), but such training is often inaccessible due to factors such as price and geographical constraints.

**Objective:**

We aim to improve the limited availability of GAVT by developing and testing a free web-based software platform (named TruVox; University of Cincinnati) that would combine real-time feedback about the user’s voice with structured vocal pitch exercises for transfeminine people.

**Methods:**

The current publicly accessible TruVox prototype focuses on vocal pitch training with 5 structured exercises that provide real-time pitch visualizations as well as supporting videos and text. It was tested in 2 evaluation stages: initial remote usability evaluations and a later single-session in-person evaluation with 21 transfeminine participants under the supervision of 2 researchers. In remote evaluations, participants reported bugs and usability issues that were iteratively addressed. In the in-person evaluation, participants tested the final software prototype and filled out the System Usability Scale, then performed 10 repetitions of different exercises to gauge performance improvement with practice. They also filled out the Intrinsic Motivation Inventory for each exercise.

**Results:**

The System Usability Scale score had a mean of 79.8 (SD 12.8) on a 100-point scale, Intrinsic Motivation Inventory scores were high (eg, interest/enjoyment over 11/14), and exercise performance significantly improved in all but 1 exercise (*P* values ranging from below .001 to .095). As qualitative feedback, participants requested to be able to use the software without much preparation and suggested several desirable future features, such as performance tracking and goal-setting.

**Conclusions:**

While the pitch training module should not be considered a complete GAVT package, TruVox represents a promising foundation for further GAVT software because it was perceived as usable and motivating and allowed participants to improve their exercise performance. To our knowledge, TruVox is the first GAVT software that combines real-time voice visualization with structured exercises, and this study represents the first quantitative human subjects evaluation of GAVT software. In the future, TruVox will be expanded with additional modules such as resonance training, then tested in longer-term trials.

## Introduction

### Voice-Gender Incongruence and Gender-Affirming Voice Training

Transgender and gender diverse people (hereinafter abbreviated as transgender people) experience a significantly lower quality of life than the general public [1], often due to poor mental health and low self-esteem associated with gender dysphoria [2]. One cause of dysphoria is voice-gender incongruence: distress because the person’s voice does not match the way their gender is perceived by themselves and others—for example, transgender women with low vocal pitch. Such voice-gender incongruence negatively affects quality of life for all transgender people [3,4]. However, it seems to be particularly an issue for transfeminine people (ie, transgender women and nonbinary people who were assigned male at birth) because hormone replacement therapy, which is commonly used by transgender people, does not affect transfeminine voices at all [5,6].

Voice-gender incongruence can be reduced using surgery, which can increase average voice pitch and perceived femininity in transgender women [7,8] as well as decrease average voice pitch in transgender men [9]. However, surgery is expensive, patient satisfaction is inconsistent [10], and there can be negative effects on other acoustic measures such as loudness and frequency range [7,8]. As a result, many transgender people are unaware that voice surgery is an option, and many of those who are aware of it would prefer a more noninvasive method [6].

The state-of-the-art noninvasive method for transgender voice modification is gender-affirming voice training (GAVT), which involves structured exercises that target different voice aspects and can be practiced over time to habituate a new speech pattern. Such GAVT is commonly provided by speech-language pathologists (SLPs) or transgender people with singing or music backgrounds and has been found to improve subjective self-perception of voice [11,12], others’ perception of voice [13], and objective acoustic measures such as pitch, resonance, and intonation [12,14-16]. While GAVT was historically mostly done in person, teletherapy via Zoom (Zoom Video Communications) and other platforms has become increasingly popular [17], increasing accessibility.

Nonetheless, GAVT accessibility remains limited for many transgender people due to geographical constraints and a shortage of experts qualified for GAVT [18,19]. Even when GAVT is available, it is generally expensive (often over US $100/hour in the United States), requires many sessions (usually 15-25 visits [16]), is frequently not covered by health insurance [20,21], and is limited by psychological barriers such as anxiety, shame, and perceived provider bias.

### Software Tools for GAVT

Given the limited accessibility of GAVT, there is an important opportunity for software that could help transgender people work on their voice by providing structured exercises, voice feedback, and other information. The software could be used as “homework” between expert-led GAVT sessions or even as a basic independent tool when human GAVT providers are not available. Such software is already used in other voice and communication training protocols besides gender affirmation [22-24] and is used for other conditions such as attention-deficit/hyperactivity disorder [23], obesity [25], and depression [26], so it should be applicable to GAVT. Indeed, such software has been praised as a promising research direction by the World Professional Association for Transgender Health [27]. Furthermore, transgender people are generally open to software solutions for other goals, such as improving adherence to HIV prevention strategies [28] and reducing minority stress [29].

The idea of science-backed GAVT software has existed since at least 2018 [30], and GAVT providers already use generic apps such as pitch monitoring tools (not originally intended for transgender people) to augment GAVT sessions, with positive results [31]. Additionally, several studies have studied the attitudes and needs of transgender people regarding GAVT software [6,30,32-35]. These studies have found positive attitudes and identified desirable features of such software: for example, it should provide visual feedback about the user’s voice (eg, a pitch graph) while the user talks, combine it with exercises that are tailored to the user’s voice goals, and track the user’s improvement over time. Unfortunately, the state of the art in GAVT software is limited, and existing tools have not achieved widespread acceptance.

To our knowledge, the first science-backed GAVT app was Project Spectra [36], which implemented only limited functions before development stalled indefinitely. Another app, Attuned [37], consists primarily of text and video, with a pitch tracker added recently but not combined with any structured exercises, and no user studies have been conducted. Other than these 2 science-backed apps, some basic GAVT apps, such as EvaF.app and Christella VoiceUp, exist but have not been systematically evaluated and do not appear broadly accepted by transgender people [6,38]. Additionally, a few studies have explored other aspects of voice software for transgender people unrelated to GAVT (eg, automated gender classification and speaker verification [39] and automated voice changers for internet-based communication [40]), but these are not directly relevant to the current work.

### Contribution of This Paper

This paper presents a prototype of TruVox: a free web-based GAVT platform that combines real-time visual voice feedback with structured voice exercises specifically for transfeminine people. While it is in the long term intended to target multiple aspects of voice, it currently focuses primarily on vocal pitch. Though transgender people correctly emphasize that excessive focus is placed on pitch in GAVT [6,30,36,41], pitch is nonetheless one of the main contributors to voice gender perception [42,43] and is easier to understand by laypeople than other contributors such as resonance [6,41]. It was therefore considered an appropriate first step, given the limited state of the art, with a vocal resonance module to be added later. Furthermore, while transmasculine people are also interested in GAVT, we chose to initially focus on transfeminine voices because the voice is affected by hormone replacement therapy in transgender men but not transgender women [5,6], as transgender women appeared most interested in GAVT in our previous work [6,17], and as feminizing GAVT has been more thoroughly studied than masculinizing GAVT [27,44,45], making software development easier.

TruVox is currently publicly accessible [46], and its source code will be made public shortly. A much earlier prototype was presented as a conference paper and poster, but included fewer than half the current features and was only evaluated with limited qualitative interviews [47,48]. Prior papers on Project Spectra and Attuned focused extensively on the process of community-based design [36,37] and critical discourse analysis [38] of GAVT software, but provided only limited software features and did not conduct systematic evaluations. Though that research is valid and important, our paper aims to provide a complementary view by presenting specific pitch exercises and supporting materials, then evaluating them with transfeminine users to quantify perceived usability, users’ intrinsic motivation, and exercise performance in a single-session study. It represents, to our knowledge, the first systematic evaluation of GAVT software that incorporates real-time feedback about the user’s voice, and serves as a basis for future multisession trials of such software that could also evaluate long-term outcomes such as perceived voice femininity.

### Researcher Positions and Terminology

The principal investigator, coauthor VDN, is a transgender femme who experienced GAVT as a client and has a background in serious games for motor rehabilitation. VSM, MEW, RLG, and TM are cisgender women and SLPs with experience in transgender research. SRW, OJ, XW, AB, TD, and RK have backgrounds in computer science or engineering and no prior experience with transgender research; RK is a transgender woman, SRW did not disclose their gender identity, and the others are cisgender men.

This paper uses identity-first rather than person-first language due to our team’s and participants’ preferences. We respect others’ rights to their own preferences.

## Methods

### Overview

This section is divided as follows. We first present key features of the TruVox software as of January 2025. We then present a first remote evaluation that was carried out to gauge overall usability and identify significant usability issues. Finally, we present a later in-person evaluation that was carried out to gauge overall usability, users’ intrinsic motivation in different exercises, and users’ performance in the exercises.

### Key Software Features

#### Overview

TruVox is compatible with most standard computer browsers, but does not currently have a mobile version. It is intended for speakers of American English, though many aspects are expected to apply to other dialects or languages. The main page [46] shows a welcome message, offers a tour, and lets users navigate further to pitch training, pitch and volume training, assessment, help, videos, and an about page.

The vocal pitch calculation algorithm used throughout TruVox is based on code from University College London [49]. It was validated during development by first playing pure tones at different frequencies, then playing recordings of the Rainbow Passage (a standard text in speech analysis) read by 16 transfeminine people before and after GAVT (obtained via our university’s GAVT Clinic). Mean pitches calculated by TruVox were compared to those calculated by Praat (University of Amsterdam), a popular open-source software package for speech analysis [50]. For pure tones, the difference between the tone and TruVox’s mean pitch was below 5 Hz. For Rainbow Passage readings, the mean difference between mean pitch in Praat and TruVox was 15.4 (SD 10.7) Hz, but this was found to be mainly due to Praat having better background noise rejection, so the algorithm was retained for this version of TruVox. Notably, all pitch calculations are done on the user’s computer, with no audio data sent through the internet. This allows TruVox to even function if the user is not connected to the internet, and was requested in our prior interview study [6] as a method of protecting user privacy.

#### Pitch Exercises

As its key feature, TruVox has 5 pitch exercises that all use the same core pitch visualization graph. The graph plots the user’s current and recent pitch values as a function of time as the user talks. It simultaneously shows a target pitch curve for the user to try to match, with the target type depending on the exercise. If the user’s pitch is close enough to the target pitch, the target curve changes color to indicate success. A start or stop button allows voice recording to be activated or deactivated, and a retry button clears the graph; these buttons are present in all exercises.

The exercises, listed roughly from easiest to hardest, are Constant, Chanting, Stair, Human Curve, and Heteronyms. They are described below in more detail than in many human-computer interaction papers due to the limited state of the art in GAVT software.

**Figure 1:**
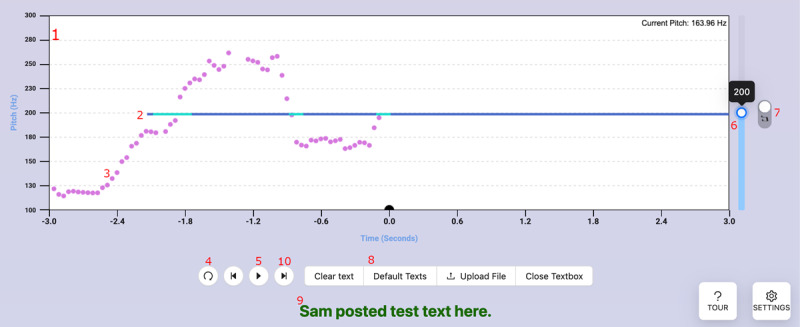
Screenshot of the Constant exercise, with elements annotated. The (1) graph shows pitch in hertz, and the user tries to match the (2) target pitch with (3) their own pitch curve. The target pitch curve changes color when the user’s pitch is close to the target. The user can (4) restart or (5) start or stop the exercise at any time, and can adjust the target pitch with a (6) slider. Next to the slider, (7) a button plays a pure tone at the target pitch for reference. The user can make whatever vocalizations they want, but can optionally (8) select from several different texts that are then (9) displayed below the graph. If the user selects a text with multiple sentences, they can use the (10) previous or next buttons to move between sentences.

The Constant exercise (Figure 1) is relatively simple: it asks the user to match a constant target pitch (presented as a horizontal line) while making any desired vocalizations. The target is 200 Hz by default (a stereotypically “feminine” pitch [16]) but can be adjusted with a slider, and a listen button allows the user to play a pure tone at the selected target. Users can also enable text that they can read during the exercise: they can select from several standard texts (Rainbow Passage, Grandfather Passage, and Harvard Sentences —all standard texts used in speech training and analysis) or type or upload their own text. The intended goal is for users to maintain an average pitch “near” the target pitch while reading, using the real-time display to adjust their voice.

The Chanting exercise also asks the user to match a constant pitch, but does so in 3 stages. The user is first instructed to hum at a constant target pitch (again 200 Hz by default, presented as a horizontal line). Once the user is sufficiently close to the target for 1 second, the exercise automatically advances to the second stage, where the user is shown a short phrase and instructed to chant it at the target pitch. When done, the graph turns blank and the user is instructed to say the same phrase at a normal conversational pace. Once the user finishes, the exercise displays the mean pitch during the final (no-feedback) stage and allows the user to restart the exercise. Users can cycle between phrases using next or previous buttons. The exercise has 4 manually selectable “difficulty levels,” which differ according to the presented phrases: the easiest level has resonant, easy-to-chant phrases (eg, “Mary made me mad”) while the hardest level has nonresonant phrases that are harder to chant (eg, “No-one knew Tim too well”). Similar exercises are described in GAVT literature [44], and specific phrases are taken from the literature as well [51]. This exercise is intended to help the user carry over their target pitch from simple vocalizations (easier to do at a target pitch) to connected speech.

**Figure 2:**
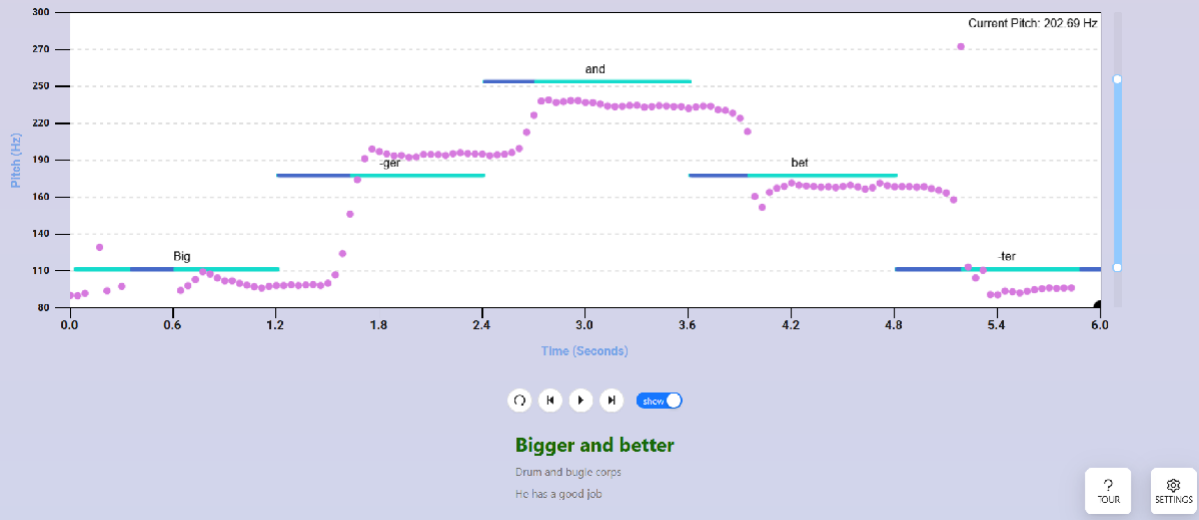
The Stair exercise, which asks the user to match a series of 5 pitch targets. As in the Constant exercise, the user’s pitch is by default shown in pink, while the target pitch curve is shown in blue. The target pitch is adjustable with the slider on the right. A show or hide button below the display optionally shows a 5-syllable phrase for the user to speak while matching the 5 pitch targets, and users can move between phrases with next or previous buttons. See Figure 1 for detailed annotation of basic elements.

The Stair exercise (Figure 2) is more challenging in that it asks the user to match 5 consecutive pitch targets: a low, medium, high, medium, and low pitch. By default, these are set to 110, 155, and 200 Hz to move from a stereotypically “masculine” pitch to a stereotypically “feminine” pitch [16] and back, but both the low and high targets can be adjusted with the slider, with the medium target automatically set midway between low and high. Again, the user can make any desired vocalization, but a button optionally displays various premade 5-syllable phrases to be spoken for the exercise, with each syllable at a different pitch target. The idea for this exercise was gained during our previous interview study, which, among other things, asked transgender people to describe exercises they experienced in GAVT [6]. Its purpose is to train vocal flexibility and prosody.

**Figure 3:**
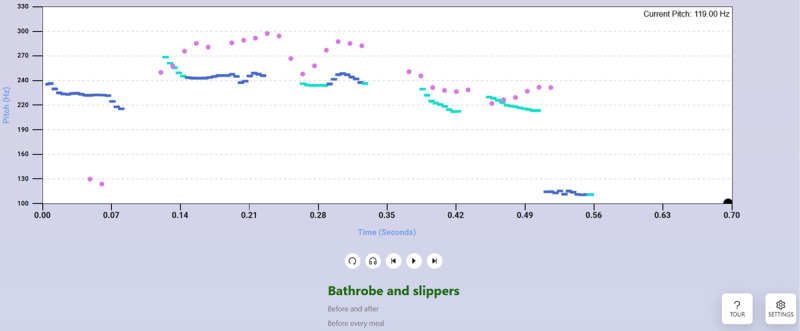
The Human Curve exercise, which asks the user to match the pitch curve of a human “model” while speaking a target phrase. As in the Constant exercise, the user’s pitch is by default shown in pink while the target pitch curve is shown in blue. A listen button (which looks like headphones) below the display lets the user listen to the actual speech recording the pitch curve is derived from, and users can move between phrases with next or previous buttons. See Figure 1 for detailed annotation of basic elements.

The Human Curve exercise (Figure 3) asks the user to match the pitch curve of a previously recorded human speaker (“vocal model”) speaking a 2- to 5-syllable phrase (manually selectable). A listen button below the graph lets users play the recording of the vocal model speaking the current phrase, and next or previous buttons let users cycle between phrases. At this time, 9 vocal models can be selected from the settings menu; they are all cisgender women and native speakers of Mainstream American English. Vocal models 1-7 are aged 20-30 years, model 8 is in her early 50s, and model 9 is in her late 60s. Eight models are White, while the remaining one is biracial (African-American and White). This exercise was developed to train natural feminine intonation patterns through imitation.

The Heteronyms exercise differs from the others in that the user is not expected to precisely match a target pitch or even intensely practice the exercise—instead, it is intended to teach users about upward and downward intonation because upward intonation is correlated with perceived voice femininity [27,43]. The exercise does this by presenting pairs of heteronyms: pairs of words that are spelled the same but pronounced differently, with stress on the first versus the second syllable. An example heteronym is the word “console.” If the stress is placed on the first syllable, it is a noun; if the stress is placed on the second syllable, it is a verb. TruVox presents the word below the pitch graph and capitalizes the stressed syllable. The pitch graph then shows a pitch curve of a vocal model speaking the word; these models are the same as those in the Human Curve exercise, and the user can again listen to the recording with the listen button. Users can switch between the 2 variants of the word with a button. Additionally, the user can switch to a sentence display. In this case, TruVox presents an example sentence using that word, the pitch graph shows a pitch curve of the vocal model speaking the sentence, and users can switch between the 2 variants of the word. Example sentences for the word “console” are “They bought a new CONsole” and “The doctor will conSOLE his patient.” The idea for this exercise was again gained during our previous interview study [6].

In all 5 exercises, a settings button next to the pitch graph allows users to modify several options: the pitch range (adjustable between 50-600 Hz), time scale (from 2.5 to 15 s), pitch display unit (Hz or musical notes), color theme (default, dark, or colorblind), and the speed with which the pitch indicator moves from left to right (from very slow to very fast). There is also a toggle to automatically start an exercise when speech is detected by the microphone (as opposed to starting manually with the start or stop button), with an adjustable volume threshold for starting. Finally, an “advanced features” setting allows rudimentary performance tracking to be activated, which was used in the in-person evaluation (presented later). Below each exercise is a “How to use” section that provides text instructions for the exercise, an embedded demonstration video (see next section), and a description of the settings.

#### Other Features

While the pitch exercises represent the core part of TruVox at this time, several other features are available.

The Pitch and Volume module contains the same Constant and Stair exercises described above, with the graph again showing pitch on the y-axis. However, the graph now also shows the volume (loudness) of each recorded sound sample. Each sample is marked as quiet, medium, or loud, and the user can select whether they want to visually indicate the volume of each sample with color (blue=quiet, purple=medium, and red=loud), dot size (small=quiet and large=loud), or both. This feature was added because participants in our previous interview study stated that they often have trouble simultaneously speaking loudly and maintaining their desired pitch [6]. It has not been extensively developed at this time and serves mainly as a demonstration of visualizing multiple voice aspects simultaneously.

The videos module contains 3 videos to help users warm up their voice (breathing, resonance, and vocal stretching) as well as demonstration videos of all 5 pitch exercises. The warmup videos were recorded with a professional voiceover and partially show coauthor RLG demonstrating warmup motions. The demonstration videos were recorded and narrated by coauthor VDN using screen sharing functionality in Zoom, with the TruVox display taking up most of the screen and video of VDN visible in the top right. The more amateur nature of demonstration videos is because they were rerecorded several times during development as features were added and visual elements were changed.

The assessment module first asks the user to hold an /i/ sound for 5 seconds, then read the Rainbow Passage. It then calculates the user’s mean pitch in both vocalizations. Further assessment features (eg, pitch range and resonance) are planned for future versions.

Finally, the help module contains background information about voice as well as text descriptions of different modules and embedded exercise demonstration videos.

### Evaluation Summary

TruVox was evaluated in single-session studies carried out both remotely and in person, with the remote evaluations carried out in 2 consecutive stages. Details of the evaluations are presented in the next 2 sections (first remote and then in-person), and Table 1 summarizes key characteristics of each evaluation. All evaluations used a single-arm design where all participants in the evaluation experienced the same study protocol with the same software.

**Table 1:** Key characteristics of consecutive TruVox evaluations.

	Remote evaluation		In-person evaluation
	First stage	Second stage	
Data collection timeframe	Spring 2024	Fall 2024	November 2024 to January 2025
Goal	Identify issues and gather feedback	Verify usability and gather feedback	Verify usability, user motivation, and exercise performance
Outcome measures	Qualitative feedback only	SUS^a^, qualitative feedback	SUS, Intrinsic Motivation Inventory, exercise error
Participants	13 completed	8 screened, 6 completed	26 screened, 21 completed
Overall protocol	Participants use the software alone and are then interviewed	Participants use software alone, give open-ended written feedback, and fill out the SUS	Participants use software in the laboratory, fill out the SUS, perform 10 repetitions of each exercise to measure exercise error, and periodically fill out the Intrinsic Motivation Inventory
Main statistical tests	None	SUS over 70?	Is SUS over 70? Is exercise error lower in later repetitions?
Already published?	Yes [47,48]	No	No

^a^SUS: System Usability Scale.

### Remote Evaluation

#### Overview

The goal of the remote evaluation was to identify key usability issues to be addressed and to gauge overall usability in a quick and convenient manner without requiring participants to come to our laboratory. It was carried out in 2 stages.

#### First Stage

The first evaluation stage was carried out in spring 2024 with an early version of TruVox that only had 3 pitch exercises (Constant, Stair, and Human Curve), a less polished visual presentation, no assessment module, no videos, no introduction page (the current help page acted as the default introduction page), and only 7 of the current 9 vocal models for the Human Curve exercise.

This prototype was first presented to 5 transfeminine participants. A link to the web page and an informed consent form were provided electronically, and participants were asked to interact with the software for 15-20 minutes on their own computer. After that, participants engaged in a brief interview with coauthor VDN to discuss usability issues and general impressions. The results of this stage were purely qualitative and were published in the prior conference paper [47], so this stage is not described further.

Additionally, the prototype was presented to 8 transfeminine participants to specifically gauge their reactions to the 7 vocal models in the Human Curve exercise. Methods and results were presented as a conference poster [48]; in brief, participants generally had positive reactions but requested a broader range of ages and dialects, some transfeminine vocal models, and some models with lower pitches.

#### Second Stage

The second stage was carried out in fall 2024 and involved a version of TruVox similar to the one described in the Key Software Features section—no major functional changes were made after this stage, only usability improvements such as reducing unnecessary space, rerecording videos, editing the help page text, and rearranging elements on the pages.

Eight people were recruited for this study via a Discord (Discord Inc) server for transgender people in Ohio and a Health Communication Disorders Clinic at the University of Central Florida. Two did not complete the protocol (did not return the forms), leaving 6 participants: 5 transgender women and 1 transfeminine xenogender participant. All 6 were native speakers of American English and were aged 21, 26, 29, 30, 33, and 34 years. Three reported previous GAVT experience with a human expert. The sample size was chosen because a simple guideline suggests that 5 participants are sufficient to identify most usability issues in first prototypes [52].

Participants were again electronically sent a link to TruVox and an informed consent form. They were given a list of 14 website components (introduction, tour, Constant exercise, settings menu, Stair exercise, Chanting exercise, Human Curve exercise, Human Curve—specifically vocal models, Heteronyms exercise, assessment page, help page and help sections, videos, about page, and any additional comments) and asked to provide open-ended written comments about each component. They could test the components in any order they desired and could write down their thoughts either while testing each component or at the end. Furthermore, they were asked not to consider the Pitch and Volume exercises as they were incomplete. Similarly, they were told that resonance exercises would be available in a future version because vocal resonance is considered important by transfeminine people [6,41], and we expected that participants would criticize TruVox if resonance was not acknowledged. Finally, they were told that performing the exercises may cause vocal strain or fatigue or psychological discomfort (also noted on the consent form) and that they should feel free to take a break or discontinue participation if this occurs.

After providing open-ended feedback about the components, participants filled out the System Usability Scale (SUS) [53] to gauge the perceived usability of TruVox as a whole. SUS consists of 10 items that are all 5-point Likert scales and are combined into a single score by first reversing the negative items, then scaling the sum from 0 to 100. As a common SUS benchmark, a score of 70 is commonly considered “acceptable” for a first prototype [54]. Thus, the SUS score was the primary outcome measure, and we planned to continue iterative development and usability testing with additional participants if TruVox did not reach an SUS score of 70 in this stage.

Participants were not monitored while using the software or filling out the questionnaires, though a single reminder was sent if they did not return the forms within 72 hours. Blank copies of the informed consent form and full questionnaire provided to participants are available via the reference citation in the Data Availability section.

### In-Person Evaluation

#### Participants

Study advertisements for the in-person evaluation were distributed to 2 Discord servers and a Facebook group for transgender people in southwest Ohio, a GAVT clinic run by University of Cincinnati Health (which coauthor RLG is affiliated with), the student mailing list of the University of Cincinnati College of Engineering and Applied Sciences (which coauthor VDN is affiliated with), and student and employee LGBTQ+ (lesbian, gay, bisexual, transgender, queer, and others) groups at the University of Cincinnati. Each advertisement included a brief description of this study’s purpose and procedure, a screenshot of TruVox (but no web link), emphasized that participants would receive gift cards but would be unlikely to see voice improvements due to participation, and directed potential participants to contact VDN by email. Readers were also encouraged to forward the advertisement to others who might be interested. Once potential participants contacted VDN by email, VDN answered any questions and verified if participants met inclusion or exclusion criteria (aged 18+ years, assigned male at birth, identify as transgender, native American English speaker, have sufficient hearing and vision, have no diagnosed speech, language, or hearing problems besides voice-gender dysphoria, no neurological issues with larynx, and no prior voice feminization surgery). Computer or internet literacy was not used as a formal inclusion or exclusion criterion, though it was likely an implicit one. If participants fulfilled the requirements, they were scheduled for a session.

A total of 26 people contacted VDN about this study. One did not meet the inclusion criteria, while 4 stopped responding to emails before scheduling a session, resulting in 21 participants who all completed the in-person session. Further, 16 were transgender women (she/her or she/they pronouns), 3 transfeminine nonbinary or transgender femmes (she/they pronouns), 1 nonbinary (they/them pronouns), and 1 uncertain of gender (he/him pronouns). They were aged a mean of 30.2 (SD 8.0) years, minimum of 19 years and, maximum of 42 years. Furthermore, 19 were White, while 2 were biracial. A sample size of 21 is higher than or similar to most formative studies of GAVT software and transgender-related software, which mostly use 4-20 participants [6,30,31,33-35]. The first participant completed their session in late November 2024, while the last participant completed it in late January 2025.

All participants were native speakers of American English; 20 reported their dialect as Midwestern or Cincinnatian, while 1 reported it as Standard American English. Dialect data were collected because our previous work suggested that the suitability of GAVT exercises may vary between dialects [6]. When asked about previous experience with voice training, 12 reported having attended GAVT sessions with a professional, 7 reported no professional GAVT but some self-guided GAVT, 1 reported no GAVT but previous singing training, and 1 reported no experience. This information was collected because we expected that reactions to GAVT software would depend on prior GAVT experience [6].

#### Study Protocol

The sessions were carried out in a quiet room on the University of Cincinnati campus by coauthors VDN and MEW, with VDN present at all sessions and MEW present at 16 of 21 sessions. TruVox was presented on a laptop (Lenovo LEGION Y920 Notebook with 17.3” screen), with the participant’s voice input to TruVox via the laptop’s built-in microphone. Throughout the sessions, a video of the screen was also taken using screen capture software, together with voice recordings, so that any usability issues could be easily tracked. To increase confidentiality, all video and audio were recorded locally to the laptop and not automatically backed up to a cloud server.

The participant filled out a demographics questionnaire (see previous subsection) while audio and video recordings were started. They were also explicitly made aware that vocal strain or fatigue or psychological discomfort may occur due to performing the exercises, and that they were free to take breaks or discontinue participation if necessary. Throughout the session, the experimenters also monitored participants for discomfort, occasionally suggested they take breaks to reduce vocal fatigue, and provided disposable water bottles if needed.

In the first half of the session, the participant completed a usability evaluation. If MEW was present, she conducted this evaluation while VDN left the room for the duration of the evaluation unless she was temporarily needed for technical support; if MEW was absent, VDN conducted this evaluation. As in the remote evaluation, participants were given a list of website components and were encouraged to test TruVox in approximately that sequence, though they could deviate if desired. As in the remote evaluation, they were told not to test the Pitch and Volume module and that a resonance module was in development. They were encouraged to provide open-ended spoken comments and were regularly prompted for feedback with general open-ended questions (eg, “What did you think?”). If participants asked for clarification about how to do something, the researchers told them to try and find it through the information available in TruVox, because we wanted to see if TruVox was usable without expert guidance. Participants were further instructed not to perform too many repetitions of each exercise during this stage, with the explanation that there would be a formal exercise component afterward. At the end of the usability evaluation, participants completed the SUS [53].

The second half of the session was conducted by VDN, with MEW (if present) providing support. In the second half, the participant completed 10 repetitions of each of 7 exercises based on Constant, Chanting, Stair, and Human Curve exercises. Each exercise was first demonstrated 1-2 times by VDN, and the participant then performed the repetitions with no further familiarization. These exercises were:

- Constant—/i/: participants held an /i/ sound at a target frequency of 200 Hz in the Constant exercise for 5 seconds.
- Constant—Rainbow Passage: participants read the second sentence of the Rainbow Passage (“The rainbow is a division of white light into many beautiful colors.”) at a target frequency of 200 Hz in the Constant exercise.
- Chanting: participants completed the Chanting exercise at level 1 and target frequency of 200 Hz with the phrase “Mary made me mad.”
- Stair—/i/: participants held an /i/ sound at target frequencies of 110, 155, and 200 Hz in the Stair exercise, with the duration of each repetition being 6 seconds. Automatic start was enabled for both Stair exercises to help participants better match the timing.
- Stair—bigger and better: participants said the phrase “bigger and better” at the same target frequencies in the Stair exercise, with each syllable at a different target (“Big-ger and bet-ter”) and the total duration being 6 seconds.
- Human Curve—model 1: participant said the phrase “bathrobe and slippers” while trying to match the pitch curve of vocal model 1 at maximum pitch indicator speed. Automatic start was disabled, and participants were given the mouse so they could start the exercise and listen to the model recording at their own pace; this preference was identified in pilot trials.
- Human Curve—self-selected model: the participant previewed all the vocal models and was told to select one they liked, with no further specific clarification. They could not select model 1 again. They then performed the same exercise as the previous one with the new vocal model.

In each exercise, a repetition was redone if the participant clearly made a mistake (eg, coughing or laughing midrepetition). The exercises were performed in a fixed order as described above; while we considered randomization, we ultimately chose an order that roughly corresponds to increasing exercise difficulty because participants may not be able to perform the harder exercises at all without experience with simpler ones. A target of 200 Hz was used as a stereotypical “feminine” pitch [16]. We originally considered providing a personalized target for each participant, but eventually chose not to do so because there is not yet a clear rule for setting participant-specific goals, and this may make data analysis more complex. We also considered a lower target in the gender-neutral range because this is sometimes recommended to reduce vocal strain in early GAVT stages [27], but decided against it because participants in pilot trials did not show major strain.

Participants filled out the Intrinsic Motivation Inventory (IMI—same 8-item version as in our previous work [55,56]) 4 times: after the Constant exercises, after Chanting, after the Stair exercises, and after the Human Curve exercises. Each time, they were instructed to rate their experience with that exercise type (eg, both Human Curve exercises). The IMI was selected because our previous studies found that it can identify differences in subjective reactions to somewhat similar tasks. After completing the last exercise, participants were given the US $20 gift card, asked for any final feedback, and thanked for their participation.

Blank informed consent forms and questionnaires are provided via a reference citation link in the Data Availability section. The same link also contains a copy of the experimenters’ in-person session checklist that was written for internal use and is published unedited.

#### Data Analysis

SUS scores were compared to the benchmark score of 70 using a 1-sample *t* test (2-tailed). For the IMI, the 8 items were converted to 4 scales: interest/enjoyment, effort/importance, perceived competence, and pressure/tension (2 items per scale). These 4 IMI scales were then compared between the 4 exercise types using repeated-measures ANOVA. As there were 4 IMI ANOVA, the Benjamini-Hochberg procedure [57] with a base α of .05 was used to reduce the false discovery rate within these 4 tests. ANOVA that were significant after the Benjamini-Hochberg procedure were analyzed with post hoc Holm-Sidak tests.

As a measure of exercise performance, the built-in TruVox code was used to calculate the mean absolute difference between the target pitch values and the participant’s pitch values over the course of each repetition. For Stair and Human Curve, 1 repetition was defined as being from the time recording was started to the time it ended automatically. For Constant /i/, it was defined as 5 seconds from the time the target pitch indicator reached the participant’s pitch indicator. For Constant Rainbow Passage, it was defined from the time the target pitch indicator reached the participant’s pitch indicator to just before the end of the spoken sentence. For Chanting, it was defined as the final phase of the exercise (when visual feedback was removed). This mean absolute difference is hereafter called the “exercise error” and reported in hertz. Each participant’s mean exercise error was calculated over the first 5 repetitions and over the second 5 repetitions of an exercise. Participants’ mean exercise errors were then compared between the first 5 repetitions and the second 5 repetitions of an exercise using 2-tailed paired *t* tests (or signed rank tests in the case of normality violations) to see if the errors decreased. The Benjamini-Hochberg procedure [57] with a base α of .05 was used to reduce the false discovery rate within the 7 paired *t* tests (for 7 exercises). Similar metrics (mean absolute difference between target and actual value) are used to measure improvement in fields such as limb motor learning [58].

Participant statements and experimenter observations are reported descriptively. To analyze participant statements during the sessions, audio (but not video) recordings were uploaded to a secure password-protected university server in a folder accessible only to VDN, MEW, and VSM, who then listened to the recordings, made notes of salient topics, and compared their impressions. No systematic qualitative analysis method (eg, thematic analysis) was used because participant comments were generally not extensive and the topics tended to repeat between participants. Following the analysis, audio recordings were removed from the server, and both audio and video recordings were backed up on 2 local hard drives in VDN’s laboratory for long-term storage.

Finally, we note that improvements in perceived voice femininity were not evaluated because these are commonly evaluated after a full GAVT regimen of 10+ sessions [12,13,16,27] and are thus beyond the scope of a single-session feasibility study.

### Ethical Considerations

All procedures were approved by the Institutional Review Board of the University of Cincinnati (#2023-0831), and were conducted per the principles of the Declaration of Helsinki. Upon the participant’s arrival, the purpose and procedure of the study were explained, and the participant signed an informed consent form. All participants were explicitly made aware of the recordings and how they would be stored and analyzed, as well as how other data would be analyzed and shared in a deidentified manner. Each participant received a US $20 Amazon gift card upon completing the evaluation.

## Results

### Remote Evaluation

As mentioned, the results of the first stage were largely qualitative and are described in prior publications [47,48]. In the second stage, the 6 participants reported SUS scores of 95, 60, 80, 77.5, 82.5, and 62.5. We thus proceeded to in-person testing after addressing significant usability issues identified in this stage.

One key usability issue listed by the 2 participants with lower SUS scores was insufficient explanation of website components for unsupervised use. For example, participants requested more tour steps, more mouseover popups for different elements, and more emphasis on optional features that were not obvious (eg, ability to automatically start the exercise or slow down or speed up the horizontal pitch indicator movement). In some cases, these components were not explained at all; in other cases, the explanations were present in either the videos or help page, but were not found by participants. This issue was echoed by the other participants.

As another issue, the participant who gave an SUS score of 60 noted that the Stair exercise “at default settings … is stressful and feels like I’m failing, impacting my desire to do this exercise negatively.” She similarly noted that the Human Curve exercise “feels impossible – no matter what I tried (including adjusting the Pitch Indicator Speed in Settings) I could not get my pitch to match the recording at all.” She continued to say that the Human Curve exercise “doesn’t feel discouraging to try like the Stair Exercise did at first, just feels ill-tuned and impossible to complete since the timing is not adjusted and feels super awkward to try and get that right.” She noted that the Heteronyms exercise “suffers from the same issues as the Human Curve exercise - feels incredibly difficult to find the right timing to match the graph and vocal model.” When queried further, the participant clarified that the challenge lay in both matching the timing (eg, intonation in the Human Curve) and matching the specific target pitch (eg, the high pitch of the default vocal model 1 in the Human Curve), and that she did not feel able to adjust the settings to an achievable target. As a possible solution, she suggested that “the site could benefit from automatically adjusting speed and pitch settings in the Human Curve and Heteronyms exercises based on the phrase and voice model rather than putting that adjustment on the trainee”. The other participant who gave a low score had similar concerns regarding timing, noting that they “seem to speak words too fast for us to get a massive bit of usage out of this tool.” Other participants generally agreed with these issues but appeared to be able to address them by adjusting the settings.

As other significant issues, participants mentioned a lack of optimization for different screen resolutions, a lack of video polish, a lack of volume control in the sound playback, an inconsistent vocal model module, a bug in the pitch calculation, and a bug with some buttons not working consistently. These issues were iteratively addressed during and after this evaluation stage.

### In-Person Evaluation

#### Quantitative Results

The mean SUS score across all participants was 79.8 (SD 12.8), with the lowest value being 45 and the second-lowest being 65. The 1-sample *t* test showed that the score was higher than the benchmark 70 (*t*_20_=28.5, *P*<.001).

IMI results for different exercises are shown in Table 2. ANOVA found that:

- Interest/enjoyment did not significantly differ between exercises (P=.26).
- Effort/importance differed between exercises (P<.001). Effort/importance in the Constant exercise was lower than in all other exercises (P<.01 for all 3 comparisons), and effort/importance in Chanting was lower than in Stair and Human Curve (P<.05 for both comparisons). Perceived competence differed between exercises (P=.007). Competence was lower in the Human Curve exercise than in Constant and Chanting exercises (P<.03 for both comparisons).
- Pressure/tension did not differ between exercises (P=.92).

**Table 2:** Intrinsic Motivation Inventory results for the 4 exercise types in the performance evaluation, presented as mean (SD) across 21 participants.

	Constant, mean (SD)	Chanting, mean (SD)	Stair, mean (SD)	Human Curve, mean (SD)
Interest/enjoyment	12.0 (2.0)	12.7 (1.4)	12.0 (2.3)	11.5 (2.9)
Effort/importance	11.2 (2.0)	12.2 (1.8)	12.9 (1.3)	12.8 (1.4)
Perceived competence	10.6 (2.5)	10.4 (3.0)	9.8 (2.9)	8.7 (3.3)
Pressure/tension	5.4 (2.4)	5.3 (2.7)	5.7 (3.6)	5.5 (3.5)

Exercise performance results are shown in Table 3. Further, 6 of 7 exercises showed a significant reduction in exercise errors (*P*<.05) between the first 5 and second 5 repetitions. When choosing a preferred model (other than model 1) in the Human Curve exercise, 4 participants chose model 2, 1 chose 3, 4 chose 4, 3 chose 5, 2 chose 6, 5 chose 7, 2 chose 8, and none chose 9. It is possible that model 9 was never selected because she is in her late 60s and thus more than 20 years older than the oldest participant.

**Table 3:** Exercise errors in the first 5 repetitions and the second 5 repetitions of the 7 exercises, presented as mean (SD) across 21 participants. *P* values are the results of tests comparing exercise errors between the first 5 and the second 5 repetitions.

Exercise type	Exercise error (Hz)		*P* value
	First 5 repetitions	Second 5 repetitions	
Constant—/i/	5.3 (4.3)	4.1 (4.0)	.005^a^
Constant—Rainbow Passage	26.8 (13.6)	20.8 (12.3)	.02^a^
Chanting	6.1 (5.1)	5.0 (5.0)	.003^a^
Stair—/i/	18.5 (9.3)	13.8 (7.2)	<.001^a^
Stair—bigger and better	13.4 (5.4)	12.4 (5.3)	.095
Human Curve—model 1	47.5 (23.7)	40.2 (17.8)	<.001^a^
Human Curve—preferred model	57.8 (32.7)	50.0 (28.4)	.003^a^

^a^Significant *P* value after Benjamini-Hochberg correction.

#### Qualitative Results

As in the remote evaluation, participants mainly raised usability issues regarding the ability to use the different exercises unsupervised. For example, opening the help page, 1 participant commented, “nobody’s going to read all that,” and requested more introductory components that would guide participants to exercises appropriate to their skill level. Similarly, 3 participants requested an overall introductory video that would introduce them to the website and explain which exercises to use. Notably, several participants stated that they did not know what the target pitch should be and requested more information about what a “feminine” pitch was. For example, 1 participant who had not done GAVT explicitly stated “I don’t have a target voice yet” and asked “how do I know what pitch to go for?” This target information was originally intentionally not emphasized due to concerns that users may be demotivated by knowing they are not in a “feminine” pitch range [6].

The Constant, Stair and Chanting exercises were generally well-received. In Stair, participants primarily expressed frustration regarding matching the timing of pitch changes; those who did not view the help page or demo videos often requested the ability to change pitch indicator speed or add an automatic start function. Additionally, 1 participant suggested an optional “delay” where the exercise would wait approximately half a second after pressing start before beginning. With Chanting, participants primarily had trouble understanding how to advance through the phases and what to do in each phase. For example, they stated that the change in phases should be more obvious (some participants did not notice it) and requested clarification about whether they should continue chanting in the final phase, whether they should speak freely at the target pitch, or whether they should speak freely at their normal pitch. Finally, when performing the Stair exercise in the second half of the session, several participants who had previously undergone GAVT noted that the lowest pitch target was difficult for them to achieve post-GAVT and that these targets should be either raised overall or adjusted for each participant.

The Human Curve and Heteronyms exercises received more mixed opinions. In Human Curve, participants generally understood the concept but experienced several challenges. Some participants had difficulty achieving the vocal models’ higher pitches, while others could reach the target pitch but could not match the cadence of the phrase. Some questioned the utility of matching a specific model’s pitch curve, with one commenting, “I don’t need to sound like someone else.” A few participants requested a larger range of vocal models, who were all cisgender women and mostly White. The Heteronyms exercise received the most mixed opinions: while it was intuitively understood by some participants, others either did not understand that the goal was not to perfectly match the target pitch curve or did not understand that they could toggle between 2 intonations or between words and phrases. One participant commented, “I don’t understand what the point of this is” regarding Heteronyms, with others expressing similar confusion.

Concerning exercise demo videos, participants generally found them informative but had mixed opinions regarding presentation. For example, several participants were happy to have the video-in-video of coauthor VDN to emphasize that the project is transgender-led, but others found it distracting. Participants also requested the videos to be more concise so that they would require minimal time to glean information. This was not a concern with the warmup videos, which were received positively with little criticism.

Concerning missing features, participants primarily requested a resonance training module, a more detailed assessment function (with more outputs besides mean pitch as well as some characterization of their voice concerning masculine or feminine ranges), the ability to play back voice recordings in exercises, better exercise performance metrics, more information about what a feminine or masculine pitch would be, and a “goal setting” module. Participants had different ideas for a goal-setting module, but the most common suggestions were a combination of automated voice analysis and questions asked by the software (eg, “how much do you know about voice?” and “how do you want to sound?”) that would then suggest specific exercises and targets or vocal models.

Concerning exercise performance, participants often noticed the exercise error shown on the screen as part of the advanced features; while the error was intended for the experimenter (who wrote it down on paper), participants had several reactions to it. Most commonly, participants expressed satisfaction when their exercise error decreased, disappointment if it increased, and sometimes asked the experimenter to show them the paper error tracking form so they could see if they had improved over time. Several participants explicitly tried changing their vocal strategies to reduce the displayed error. Notably, 1 participant expressed disappointment when she achieved an exercise error of 0.6 Hz in the first repetition of Chanting, stating that “now I’m definitely not going to get better.”

Several participants compared TruVox favorably to Voice Tools, a smartphone app by DevExtras (available on the Google Play store) that tracks pitch and indicates associated gender. Participants primarily noted that they appreciated the structured exercises and that they liked that TruVox did not “gender” their pitch. For comparison, Voice Tools has a blue-colored range for “masculine” pitch and a pink-colored range for “feminine” pitch. While TruVox does use blue and pink in its base color scheme (Figures 1-3), it does not use them to convey femininity information, and different colors are used in dark and colorblind schemes. Other apps were not brought up during sessions, and Voice Tools was likely mentioned because it is used as part of SLP-guided GAVT at the University of Cincinnati Health GAVT clinic, a participant recruitment location.

## Discussion

### Principal Results

The in-person evaluation found encouraging results. First, the mean SUS was 79.8 (SD 12.8), which is approximately a B on a scale from A (best) to F (worst) [54] and significantly surpasses our initial goal of 70. The worst scores were obtained on the items “I found the website unnecessarily complex” and “I needed to learn a lot of things before I could get going with this website,” which is likely related to qualitative observations of participants being overwhelmed by the amount of information or having trouble finding the information they needed.

Second, IMI scores were generally good (Table 2—interest/enjoyment and effort/importance consistently over 11 of 14, pressure/tension consistently below 6 of 14). While IMI results are difficult to benchmark because there are many different variants of the IMI, they can be compared to our previous studies with the exact same 8-item IMI. In a study of a 2-player game, for example, we found that, on average, interest/enjoyment was about 10 and effort/importance was about 11 [55]. In a study of a resource management scenario with difficulty adaptation [56], we found interest or enjoyment values around 10, effort/importance around 12, competence around 9, and pressure/tension around 10. Thus, participants found the TruVox exercises enjoyable, challenging, and not very stressful. Stair and Human Curve required more effort than Constant and Chanting, which was also supported by qualitative observations: several participants commented that those exercises felt harder. As a result, participants also felt less competent in the Human Curve. This was unsurprising because natural progression through GAVT exercises also tends to begin with easier exercises, similar to Constant and Chanting.

Third, participants’ performance significantly improved throughout 10 exercise repetitions in all exercises except Stair—bigger and better (Table 3). This is a promising result because it indicates that participants can learn the exercise and improve at it with a limited number of repetitions. In our opinion, the lack of statistical significance (*P*=.095) in one exercise is not very concerning because the other exercises do show improvement, there was a numerical difference in the expected direction between the first 5 and second 5 repetitions, and there are several reasons (discussed in the Study Limitations section) why performance may not show consistent improvement. The significant decreases in exercise error (ranging from about 1 Hz to 8 Hz—Table 3) are admittedly difficult to contextualize because no other GAVT software has been quantitatively evaluated, and standard SLP-guided GAVT regimens do not quantify errors on the level of individual exercise repetitions or even individual sessions. However, studies of human vocal pitch perception suggest that a pitch difference of 3-8 Hz in normal speech would be noticeable to typical adults [59,60], so the improvements observed during most of our exercises should be perceptible. As another comparison, a typical increase in mean pitch due to voice feminization surgery or a full GAVT regimen (over 10+ weekly sessions) is 25-30 Hz [7,8,12,16], which is higher than the improvements seen in our session and would be expected to generalize across all of a speaker’s utterances. It is possible that long-term practice with the software would indeed lead to generalized voice improvements (eg, perceived as more feminine), but this is impossible to estimate from single-session results because classic GAVT regimens do not quantitatively track performance on a per-session basis and thus cannot be used for comparison. Nonetheless, while a positive single-session result does not guarantee long-term improvement, we do feel that a negative result here would have indicated that the exercises are not very suitable for users.

Overall, the evaluation found TruVox to be usable and motivating and showed that participants were able to improve their performance in the exercises over time. While a full version of the software would need to target several voice aspects besides pitch and would be tested over a much longer period, the current evaluation suggests that the TruVox pitch module is a promising foundation that can be built upon for future technology-aided GAVT.

### Next Development Steps

Participants noted several possibilities for software improvement, and several other desirable features of GAVT software are already known from previous interview studies of transgender people [6,30,32-35]. In the future, we will thus expand TruVox with such features and recommend them for other GAVT software as well.

Notably, several participants requested a goal-setting module, and such a module may be developed based on prior work on individualized goal setting in GAVT [35]. Alternatively, it may be based on voice training software for applications outside GAVT, which has already used intelligent adaptation rules to set goals based on user performance [61]. We will also add user accounts and link the goal-setting module to them so that users’ goals can be stored between sessions and periodically reevaluated. The accounts will also let users visualize their voice improvements over time, which represents a potentially strong motivator [6]. The desire to track improvement was also observed qualitatively during our in-person evaluation, where participants kept track of their performance and commented on improvements. As an additional feature, we will also increase the diversity of vocal models. For example, transgender women and nonbinary people of different ages will be recruited to serve as models, and models with a larger variety of dialects will be recruited because model dialects are known to be important in GAVT [48].

We will also develop modules to target voice aspects besides pitch. As a key feature, transfeminine people often consider resonance to be more important than pitch [6,41]. We are thus developing a resonance module based on a previous laboratory-based software implementation by coauthor TM [62], and a preliminary paper about this module was recently accepted to a conference [63]. However, this preliminary study [63] suggests that a resonance module requires more explanation because quantitative metrics of resonance, such as formant frequencies [62], are not as easily understood as pitch metrics like musical notes. Users also do not know how to change their vocal resonance and need more instruction to effectively use the module—our current implementation advises users to watch about 10 minutes of instruction videos before exercising, which may conflict with the observed desires to use the software with minimal preparation. These issues will be carefully considered when further developing the resonance module and other modules, such as the currently incomplete Pitch and Volume module. That module was requested in our prior interview study [6] because transgender people often have trouble simultaneously maintaining a target pitch and target loudness, but simultaneous visualization and training of multiple aspects of a motion is a significant challenge in motor learning [64]. Similarly, our pilot resonance module tests [63] suggest that simultaneously visualizing pitch and resonance may be helpful, but this would again need to be carefully designed to avoid overwhelming users.

Separately from exercise modules, we will also improve the underlying acoustic processing algorithms. As mentioned, our current pitch calculation algorithm (based on code from University College London [49]) achieved about a mean 15.4 (SD 10.7) Hz difference from Praat [50], a popular benchmarking software. This was due to worse background noise rejection and resulted in occasional pitch estimation errors that confused users. In the future, we will thus replace the current pitch calculation algorithm with other algorithms and compare them to Praat to identify algorithms that achieve good pitch estimation, specifically in transfeminine voices—similar to coauthor VSM’s previous work that compared Praat to custom algorithms for dysphonic voices [65]. Similar work will be done to identify the most robust algorithms for the calculation of other measures, such as formant frequencies. Based on our team’s experience with remote GAVT, we believe that these algorithms will need to take the specifics of GAVT into account: many standard background noise rejection algorithms, such as those used in Zoom videoconferencing software, would not be suitable because they filter out many sounds made during GAVT exercises, such as humming and pitch glides. In addition to this noise reduction work, we will instruct users to use TruVox in quiet spaces and may add simple algorithms to warn users if excessive background noise is detected.

Finally, once multiple modules have been implemented, they will need to be combined into a complete software package that can help deliver GAVT over longer durations. If the software is used as a supplement to SLP-guided training as suggested by other authors [31], this would be relatively easy, as the SLP could periodically reevaluate the user’s voice and suggest appropriate exercises and settings. However, if the software is used as an independent GAVT tool for people who cannot or do not want to engage in professional training, as suggested by some of our past interviewees [6], it would need additional features. For example, it would need to present exercises and materials appropriate to the user’s skill level without overwhelming users with too many possibilities, and it may need a way to monitor the voice for signs of vocal strain [6,27]. Furthermore, such software would need to avoid influencing transgender voices in undesirable ways—for example, it should not make the voice conform to undesirable gender norms [38]. Nonetheless, in the long term, such efforts may lead to a complete software package that can help increase the accessibility of GAVT and consequently improve the quality of life for transfeminine people.

### Study Limitations

As our study is a formative evaluation of prototype software, several methodological choices were made due to convenience. Additionally, because the study represents (to our knowledge) the first quantitative evaluation of GAVT software, several methodological choices had to be made based on protocols from adjacent fields such as limb motor learning. Below, we describe some key study limitations.

#### Participant Recruitment and Involvement of Developers in Evaluations

Due to convenience sampling, our evaluation was conducted with mainly White participants with Midwestern dialects and a relatively narrow participant age range (19-42 years). Future studies should also include older participants and a more diverse sample to examine, for example, the effects of technological literacy on perceived usability, vocal model preferences (as preliminarily investigated in our prior work [48]), and effects of participant dialect on exercise performance, as this would allow the software to benefit a broader segment of the target population. Previous papers have particularly noted that GAVT software tends to reproduce ideals of gender as White and able-bodied, so we consider a more racially diverse sample critical for the future [38].

Similarly, for convenience in formative evaluations, the head of the development team (VDN) also served as an in-person evaluator, which introduces potential bias because she may have intentionally or unintentionally discouraged negative feedback—for example, due to participants not wanting to disappoint a developer. We originally also made this choice for easier technical support in case of unexpected bugs, as well as representation of the target population (VDN is a transgender femme). However, having a transgender developer act as an evaluator may have introduced further bias, as participants may be even less willing to disappoint a developer who belongs to the same marginalized population. In the future, we recommend having such evaluations conducted by transgender people who are unaffiliated with the development team, who identify themselves to participants as independent evaluators, and do not show an overly “medicalized” view of GAVT due to documented distrust that some transgender people have toward clinicians [6,38,41].

#### Evaluation of Exercise Performance

The exercise error metric (absolute difference between actual and target pitch) was selected based on our prior arm motor learning work [58] but may be suboptimal for voice exercises. For instance, if participants in the Human Curve exercise vocalize the same pitch curve faster or slower than the target curve, this would be considered a large error even though it would be highly desirable from an exercise performance perspective. We nonetheless used this simple metric because it is easily understood and still showed significant improvements in exercise performance, but future studies may consider other metrics that are more robust to, for example, differences in timing.

Similarly, to evaluate improvements in exercise performance, we compared the mean exercise error from the first 5 exercise repetitions to the error from the second 5 repetitions. However, exercise error does not consistently decrease due to various factors: for example, some participants exhibited vocal fatigue in later repetitions, while other participants tried out a new exercise strategy in later repetitions that did not work and consequently resulted in higher errors. Again, we used this simple comparison because it nonetheless showed significant improvements, but future studies may consider other metrics.

#### Fixed Order of Exercises and Fixed Pitch Targets

Performing the exercises in a fixed order from approximately easiest to hardest almost certainly influenced results. For example, while the Stair—bigger and better exercise was described by several participants as harder than the Stair—/i/ exercise, participants exhibited lower exercise errors in the bigger and better exercise (~12 Hz) and did not show significant differences between the first 5 and second 5 repetitions of the bigger and better exercise. This is likely because they had already performed the Stair—/i/ exercise and were able to transfer those skills to the second Stair exercise. Similarly, exercise errors would have likely been even higher in the later exercises if participants had not already performed the earlier, easier exercises. Fatigue likely also influenced results, as participants were more likely to complain about throat soreness and ask for water and breaks later in the session, potentially leading to worse exercise performance. We nonetheless believe that this is not a critical weakness, as actual GAVT would realistically introduce easy exercises before advanced ones.

Related to that issue, using fixed pitch targets (eg, 200 Hz) not adapted to the participant’s goals or abilities likely also influenced results. For example, some participants found the upper or lower targets in the Stair exercise difficult to reach. The fixed targets were chosen for simplicity, but future studies may consider personalizing the targets based on an initial voice assessment and the participant’s abilities and interests.

#### Other Considerations

Concerning the usability evaluation (which achieved an SUS score of 79.8), it is worth noting that our participants performed the evaluation almost completely “blind”: the experimenter described the concept and provided a list of components to test, but did not provide further guidance. A higher usability score may be achieved if the website is introduced by an SLP during regular GAVT sessions, as recommended by the World Professional Association for Transgender Health [27] and as done with generic voice apps in GAVT studies [31]. However, at the same time, not all transgender people would be likely to use GAVT software with SLP supervision [6], and having the software be usable independently is likely beneficial even for those who would combine it with SLP guidance.

Additionally, while all our participants felt that GAVT had value, not all of them were actively interested in improving their pitch: for example, several had already completed a GAVT regimen with an SLP and did not desire further pitch improvement, and at least 1 stated that she was more interested in improving her resonance than her pitch. Instead, these participants primarily wanted to help evaluate software that may help other transgender people. Therefore, SUS results may change if the study were conducted only with transgender people who are at the stage of GAVT, where they are improving their pitch. As an ad hoc test of this, we divided participants into those who had experienced GAVT with a professional (12 participants) and those who had not (9 participants) and used a *t* test to compare SUS scores between groups. SUS scores were 76.5 (SD 14.2) in those with professional GAVT versus 84.3 (SD 9.7) in those without (*t*_19_=−1.42, *P*=.15); while this is not significant in our small sample, we believe it may be worthy of investigation in the future. Such differences between participants may also affect outcomes other than SUS scores: for example, exercise error in the first 5 repetitions of the Constant—/i/ exercise (first exercise in the performance evaluation) was 4.1 (SD 3.0) Hz in those with professional GAVT versus 6.8 (SD 5.4) Hz in those without (*t*_19_=−1.49, *P*=.15), indicating potential effects of prior GAVT experience on exercise performance.

### Future Clinical Trials of GAVT Software

Once our software has been further expanded into a complete GAVT package (see the Next Development Steps section), it should ideally be tested in a clinical trial to evaluate its long-term effectiveness. The primary outcome metric of such a trial would be self-reported or externally perceived changes in voice femininity, which serve as primary subjective outcomes of many GAVT trials [12,13,16,27]. Such changes are commonly evaluated at the end of a full regimen of more than 10 sessions and were thus considered beyond the scope of the current first study.

As TruVox is, to our knowledge, the first systematically evaluated GAVT software, a clinical trial would not be able to compare it to other software specifically for GAVT—Project Spectra [36] and Attuned [37] have only limited functionality, while EvaF.app and Christella VoiceUp do not appear to be broadly accepted by transgender people [6,38]. In our opinion, a reasonable first clinical trial would use TruVox as daily “homework” between weekly SLP-guided sessions and compare it to either standard homework (eg, written instructions for exercises) or to a generic voice analysis app as carried out in prior GAVT work [31]. In addition to comparing changes in voice femininity, such a clinical trial should also collect qualitative feedback via semistructured interviews, as this would provide insight into, for example, what motivates GAVT clients to practice at home. Furthermore, the trial should be conducted by a researcher who was not involved in TruVox development to reduce experimenter bias. We are currently piloting such a clinical trial of the current pitch module and the aforementioned resonance module [63] over a shorter duration (4 sessions) to identify issues that need to be addressed before a longer trial.

Simultaneously, we do not believe that a clinical trial is strictly necessary before TruVox can be used by transgender people. Many transgender people already use YouTube videos, generic voice analysis tools, and other unvalidated GAVT software (eg, EvaF.app) as part of their GAVT [6,38] despite potential safety concerns, and TruVox could be immediately used by transgender people and SLPs however they desire. We are thus preparing for a public announcement and simultaneous open-source code release. A public release will also allow us to conduct observational studies of, for example, how transgender communities use GAVT software without SLP supervision or how SLPs integrate such software into their GAVT regimens.

### Extension to Transmasculine or Nonbinary Voice and Other Languages

Our current work focused on GAVT for transfeminine people for multiple reasons. Nonetheless, we acknowledge that transmasculine people are also interested in GAVT and that the reasons that led us to start with transfeminine voice are also the reasons why transmasculine voice has historically been neglected in GAVT and general research [44,45,66]. Several key components of TruVox (eg, real-time feedback about pitch, resonance, and loudness) are likely applicable to voice masculinization because transmasculine people have also expressed interest in them [6]. At the same time, a version of TruVox for transmasculine voice cannot be created by simply “reversing” the current feminizing exercises, as masculinizing GAVT exercises differ significantly from feminization exercises [45]. Nonetheless, we believe that first steps can be taken by reusing some of the exercises that would likely be applicable (eg, Constant and Stair exercises with lower target pitches), preparing supplementary material targeted at transmasculine users (eg, videos about the reused exercises presented by a transmasculine speaker), and simultaneously developing new exercises based on both academic and community sources about transmasculine voice.

Additionally, while our current work did include some nonbinary participants, TruVox is intended to train a “feminine” voice, and a future transmasculine version would likely train a “masculine” voice. However, interviews of nonbinary people suggest that they may not be interested in a “binary” voice—they may prefer to, for example, target a “neutral” vocal gender, adopt some masculine and some feminine voice features, or have the ability to vary their voice depending on the situation [6]. Such GAVT could, in the long term, be supported with a version of TruVox that includes both feminizing and masculinizing exercises, supporting materials that provide suggestions to nonbinary users, and potentially a goal-setting module (already discussed) that would be responsive to the needs of nonbinary users.

Finally, TruVox currently primarily targets native speakers of American English. While many elements of TruVox would likely apply to other dialects and languages, several changes would also be needed to support those languages and dialects. Obviously, the vocal models would need to be rerecorded, and different phrases would need to be selected. Different phrases may even be needed for different dialects, as participants in our prior interview study anecdotally suggested that phrases used in GAVT may have different intonation in, for example, British English [6] and thus may not be suitable for exercises such as Heteronyms that depend on specific intonation. More broadly, vocal indicators of gender are known to be different in other languages [67], so the optimal target values (eg, the 200 Hz) may be different for non-English speakers. However, to the best of our knowledge, there is little systematic research on the transferability of GAVT techniques between languages, and GAVT in other languages, such as German, does use similar exercises [66], so it is currently unclear how much GAVT software would need to be tailored to other languages.

### Conclusions

While it currently only offers pitch exercises, TruVox was perceived as usable and motivating by transfeminine people. Additionally, exercise performance improved over the course of 10 repetitions in all but 1 exercise, indicating that participants were able to improve their skills in these exercises with practice. As qualitative feedback, participants wanted to be able to use the software without much preparation and suggested several desirable future features, such as performance tracking and goal-setting. Based on these positive results, we believe that the current version of TruVox represents a promising GAVT software foundation that will be expanded with modules such as resonance training in the future. To our knowledge, it is the first GAVT software that combines real-time voice visualization with structured exercises, and the in-person evaluation represents the first quantitative human subjects evaluation of GAVT software. In the long term, such software may provide a free and convenient way for transgender people to access GAVT, thus reducing their voice-gender incongruence and consequently improving their mental health. Additionally, it may be broadly beneficial for anyone who wants to change the sound of their voice.

## Abbreviations

GAVT: gender-affirming voice training
IMI: Intrinsic Motivation Inventory
LGBTQ+: lesbian, gay, bisexual, transgender, queer, and others
SLP: speech-language pathologist
SUS: System Usability Scale

## Data Availability

Deidentified data from the in-person evaluation, all blank informed consent forms and questionnaires used in the study, and the experimenter checklist for in-person sessions (originally intended for internal use) are publicly available on Zenodo [68].
